# Post mortem brain temperature and its influence on quantitative MRI of the brain

**DOI:** 10.1007/s10334-021-00971-8

**Published:** 2021-10-29

**Authors:** Celine Berger, Melanie Bauer, Holger Wittig, Eva Scheurer, Claudia Lenz

**Affiliations:** 1 Institute of Forensic Medicine, Department of Biomedical Engineering, University of Basel, Basel, Switzerland; 2 Institute of Forensic Medicine, Health Department Basel-Stadt, Basel, Switzerland

**Keywords:** Magnetic resonance imaging, Neuroimaging, Brain, Temperature, Post mortem

## Abstract

**Objective:**

MRI temperature sensitivity presents a major issue in in situ post mortem MRI (PMMRI), as the tissue temperatures differ from living persons due to passive cooling of the deceased. This study aims at computing brain temperature effects on the MRI parameters to correct for temperature in PMMRI, laying the foundation for future projects on post mortem validation of in vivo MRI techniques.

**Materials and methods:**

Brain MRI parameters were assessed in vivo and in situ post mortem using a 3 T MRI scanner. Post mortem brain temperature was measured in situ transethmoidally. The temperature effect was computed by fitting a linear model to the MRI parameters and the corresponding brain temperature.

**Results:**

Linear positive temperature correlations were observed for *T*_1_, *T*_2_* and mean diffusivity in all tissue types. A significant negative correlation was observed for *T*_2_ in white matter. Fractional anisotropy revealed significant correlations in all gray matter regions except for the thalamus.

**Discussion:**

The linear models will allow to correct for temperature in post mortem MRI. Comparing in vivo to post mortem conditions, the mean diffusivity, in contrast to *T*_1_ and *T*_2_, revealed additional effects besides temperature, such as cessation of perfusion and active diffusion.

## Introduction

The validation of in vivo magnetic resonance imaging (MRI) techniques based on macroscopy and histology is crucial to reliably characterize tissue and verify pathological changes observed in MR imaging [1]. In contrast to ex-situ post mortem MRI, in situ post mortem MRI does not suffer from artefacts due to extraction and fixation of the organ, such as shrinking, gas inclusions and changes of relaxation times and tissue diffusion properties [2]. Thus, the correlation of in situ post mortem MRI with in vivo MRI on one hand, and with macroscopy and histology, on the other hand, allows interconnecting in vivo MRI to macroscopy and histology. Therefore, in situ post mortem MRI presents a unique possibility for validating in vivo imaging techniques. However, post mortem quantitative MRI is impaired by the temperature dependence of the MRI parameters, including *T*_1_, *T*_2_, *T*_2_*, mean diffusivity (MD) and fractional anisotropy (FA) [3–10].

Previous literature revealed that temperature influences the spin–lattice relaxation *T*_1_ and the spin–spin relaxation *T*_2_ due to its relation to the translational and rotational motion of hydrogen protons [11, 12]. Nelson et al. [11] proposed a fast exchange two-state (FETS) model claiming that *T*_1_ is proportional to exp(− *E*_*A*_/*k*_*B*_*T*), which describes the relation between *T*_1_ and the absolute temperature *T* with the help of the activation energy *E*_*A*_ and the Boltzmann constant *k*_*B*_. Assuming a small range of temperature (∆temperature = 40 °C), a linear relation between the relaxation parameter *T*_1_ and the temperature could be expected [11]. Further, the model predicted a small temperature dependence of *T*_2_ [4, 11].

In contrast to in vivo MRI, the temperature dependence of the relaxation times affects qualitative and quantitative images in post mortem MRI, as the tissue temperature can markedly differ from that in living persons due to the passive cooling of the deceased subject (algor mortis) [8]. Storing the bodies in a cooling chamber of 4 °C leads to varying post mortem body temperatures in the range of 4–37 °C (assuming a normal body temperature at the time of death without the presence of fever [13]), depending on the post mortem interval and the storage time in the cooling chamber [14]. Nevertheless, post mortem MRI, especially in situ MRI, offers a unique platform for performing validation of in vivo MRI techniques [15]. As post mortem in situ investigations neither require the extraction of the brain from the skull nor tissue fixation, the intact brain of a deceased subject can be studied. However, for the validation of in vivo MRI based on in situ post mortem MRI, the effect of the temperature on the relaxation parameters has to be accurately examined to correct the relaxation parameters for the temperature in situ. To date, only a few articles that investigated temperature effects on the brain in MRI [3–10, 16, 17] were published. Birkl et al. [4] examined the temperature effect on relaxation parameters of fresh post mortem brain slices by heating the slices and measuring them using a 3 T MRI scanner. Scheurer et al. [3], Zech et al. [5], Tashiro et al. [6], Ruder et al. [8], Busch et al. [9], as well as Flach et al. [10] examined the MRI parameters as a function of the core temperature determined in the rectum with a 1.5 T MRI scanner, while Tofts et al. [16] corrected the MRI parameters based on the diffusion constant in the cerebrospinal fluid of two deceased subjects with enlarged ventricular volumes. Kobayashi et al. [7] revealed the temperature dependence of the MRI parameters based on tissue contrast changes, while Zech et al. [5] investigated the effect of temperature on the relaxation parameters in situ with a 1.5 T MRI scanner by assessing the core temperature in the esophagus. However, in contrast to the brain temperature, the core temperature depends on the body mass, body integrity and the clothing of the corpse [18]. Furthermore, different temperature cooling rates have been observed for the core and the brain temperature at identical environmental temperatures [19, 20]. Thus, these temperatures do not correlate linearly during post mortem cooling, questioning the linear relation between the MRI parameters of the brain and the core temperature proposed by prior publications [5–7, 16]. Therefor to reliably assess the temperature dependence of the brain MRI parameters, quantitative MRI should be directly correlated with brain temperature. However, this has not yet been performed and represents a major research gap.

Thus, this study investigates the effect of the brain temperature on *T*_1_, *T*_2_, *T*_2_*, MD and FA of the brain in situ, as this would enable the temperature correction of post mortem quantitative MRI. The present study further aims at comparing the MRI parameter results between in vivo and post mortem conditions at in vivo temperature. This will be achieved by evaluating the temperature dependency of the MRI parameters, both including and excluding in vivo conditions. A discrepancy between these fits at in vivo temperature would indicate that further physiological processes are present and affect the MRI parameters. Such processes might be connected to the course of dying and might include the cessation of circulation and subsequent loss of perfusion, as well as ceased metabolic activities post mortem.

## Materials and methods

### Subject characteristics

All procedures conducted in this study were performed according to the national human research act (HRA, SR 810.30) and ethical standards, and additionally with a positive evaluation of the institutional review board. In this study, 16 forensic cases (age at time of death = 61.0 ± 15.4 years; 4 females, 12 males) with an autopsy order by the local prosecutor underwent an in situ post mortem MRI examination of the brain. Post mortem subjects were excluded in case of traumatic brain injury, external signs of putrefaction (green coloration of the skin, ablation and vesicle formation of the skin [21]), cranial fractures, underage at the time of death or MR unsafe bodies. Brain and cranial integrity as well as MR safety were verified by performing a CT scan (Siemens Somatom Emotion 16 slice scanner, Siemens Healthineers, Erlangen Germany) prior to study inclusion. Eleven deceased subjects were stored in a cooling chamber at 4 °C, while the remaining five subjects were stored at an average room temperature of 19 °C prior to the MRI scan. In addition to the post mortem cases, the MRI examination was conducted on four living volunteers (age = 29.5 ± 5.6 years; 4 females), to provide reference values in vivo. MR safety of volunteers was clarified with a standard MR safety screening questionnaire, further exclusion criteria for living subjects were pregnancy or fever.

### Post mortem interval and temperature assessments

**Fig. 1:**
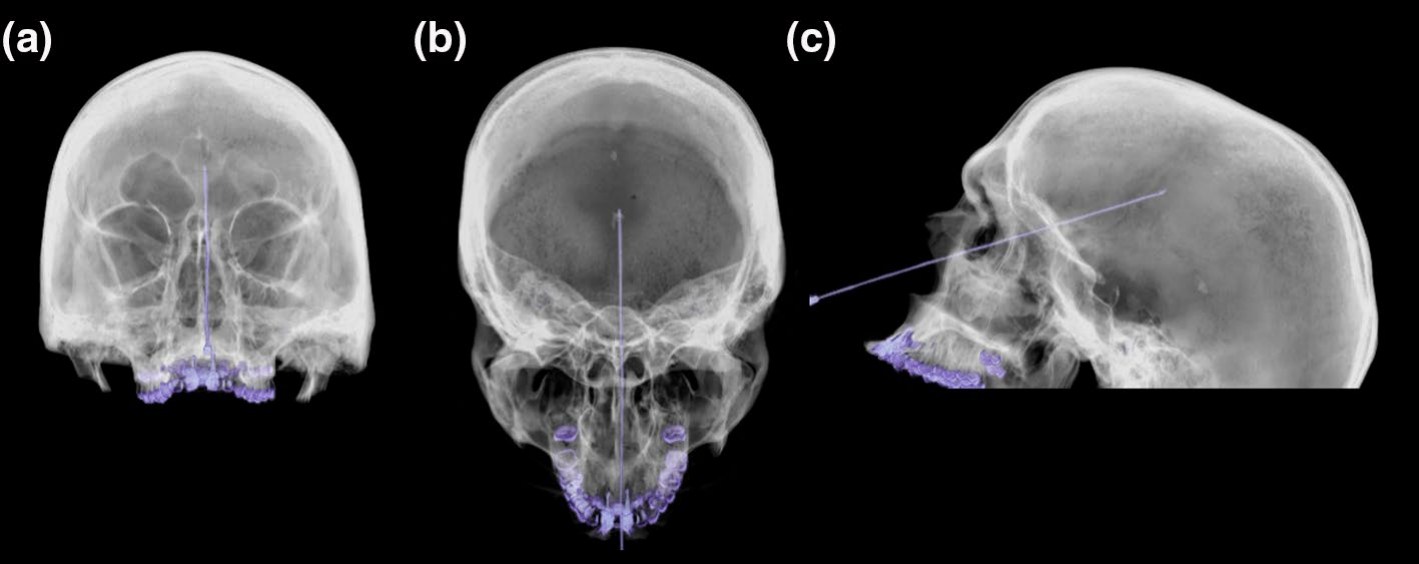
CT scan illustrating the skull in the osseous transparent metal mode of the console software (Siemens Healthineers Syngo CT 2014 A VB42B) of one post mortem subject represented in the coronal (**a**), axial (**b**) and sagittal (**c**) plane with the temperature probe placed transethmoidally

The post mortem intervals (PMI) were assessed based on Henssge’s nomogram [22] using the rectal temperature, which is the standard method for assessing the time of death used in forensic medicine. Brain temperature was assessed with a waterproof needle probe (Testo, art. no: 0628 0027, Testo SE & Co., Mönchaltorf, Switzerland) placed through the os ethmoidale along the longitudinal fissure (see Fig. 1) under CT control and removed shortly before the MRI scan due to its MR unsafe configuration. The temperature measurements were acquired with a recording interval of 10 s using a portable temperature data logger system (Testo 175 T3—Temperaturlogger, Testo SE & Co., Mönchaltorf, Switzerland). The brain temperature of the living volunteers was assumed to be 36.5 °C [23, 24].

### MRI acquisitions

During the MRI scan (3 T Siemens MAGNETOM Prisma with a 20-channel head and neck coil) the corpses were wrapped in two artefact-free body bags in supine position to prevent fluid leakage and to keep anonymity of the bodies. The following MRI protocol was always acquired in the same order in post mortem and in vivo subjects and had a scan duration of 1.5 h:

- Inversion recovery spin-echo (IR-SE) sequence with six different inversion times to quantify relaxation time *T*_1_ (TI = 30, 80, 200, 400, 700, 1200 ms, echo time (TE) = 12 ms, repetition time (TR) = 7060 ms, 40 slices, slice thickness of 4 mm, in-plane resolution of 1 × 1 mm^2^);
- Multi-contrast spin-echo (SE) sequence with 12 different TEs to quantify relaxation time *T*_2_ (TEs = 9.8, 19.6, 294, 39.2, 49.0, 58.8, 68.6, 78.4, 88.2, 98, 107.8, 117.6 ms, TR = 5720 ms, 44 slices, slice thickness of 4 mm, in-plane resolution of 1 × 1 mm^2^);
- Multi-echo gradient echo (GRE) sequence with 12 different echo times to quantify relaxation time *T*_2_* (TEs = 5.79, 10.34, 14.40, 18.46, 22.52, 26.58, 30.64, 34.70, 38.76, 42.82, 46.88, 50.94 ms, TR = 68 ms, 44 slices, slice thickness 4 mm, in-plane resolution of 1 × 1 mm^2^);
- Diffusion-weighted single-shot echo-planar imaging DTI sequence to quantify MD and FA with *b* = 2000 s/mm^2^, 64 isotropically distributed diffusion directions, 3 *b* = 0 s/mm^2^, TE = 109 ms, TR = 18,700 ms, 100 slices, isotropic resolution of 1.8 mm^3^.

### Image analysis

**Fig. 2:**
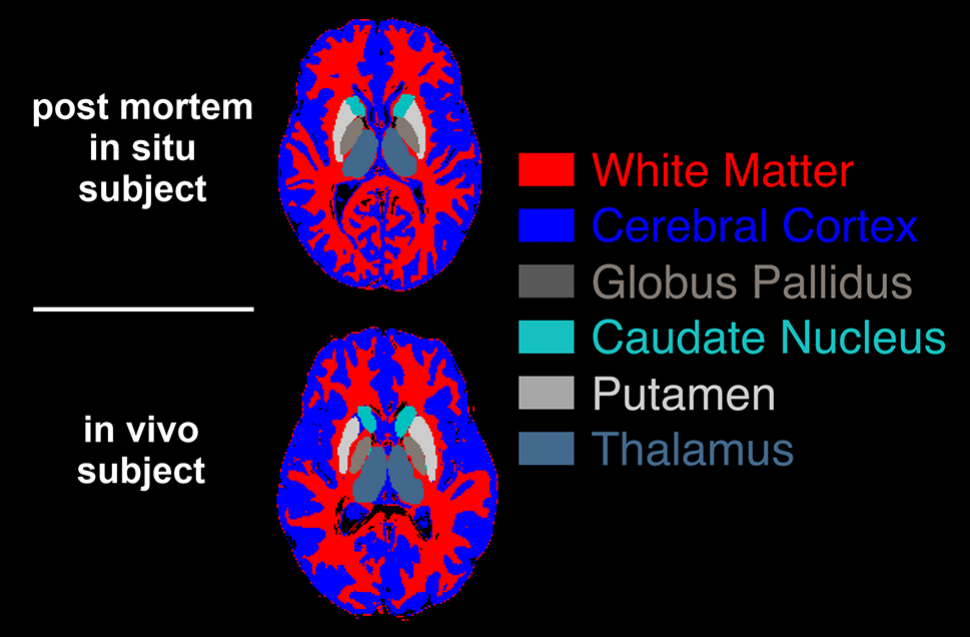
Segmentation examples of the investigated regions in one slice represented in one post mortem and one in vivo subject. The same color scheme will be used for Figs. 4 and 5 for visibility reasons

Brain extraction was performed on the *T*_1_-weighted IR-SE data with TI = 200 ms, using BET [25] of the software functional MRI of the brain software library (FSL) 6.1 (Analysis Group, FMRIB, Oxford, UK) [26]. Further, FSL’s FAST [27] was used to generate the white and gray matter masks of the *T*_1_-weighted IR-SE data. The deep gray matter mask (including nucleus accumbens, amygdala, hippocampus, globus pallidus, putamen, caudate nucleus, and thalamus) was generated using FSL’s FIRST [28]. For further analysis, the deep gray matter subregions globus pallidus, putamen, caudate nucleus, and thalamus were segmented separately using FSL’s FIRST (see Fig. 2). Voxels revealing partial volume effects on the FSL partial volume maps [27] (values below 1 in the partial volume maps) were excluded for further calculations. In addition, partial volume effects were further avoided by thresholding the maps of the MRI parameters using the Otsu method [29, 30], which maximizes the interclass variance of the voxels with and without partial volume effects. In the case of poor CSF segmentation, manual segmentation was performed on the MRI parameter maps.

SE and GRE were registered to the IR-SE data using FSL’s FLIRT [31, 32], which allowed the subsequent application of white matter, cerebral cortex and deep gray matter masks to SE and GRE datasets. *T*_2_ and *T*_2_* were computed voxel-wise using a two-parameter mono-exponential single decay fit [33], while the relaxation time *T*_1_ was calculated voxel-wise using a biexponential fit with three parameters (${M}_{0}$, *p*, *T*_1_) and *T*_2_ of the corresponding voxel $\left( {S = M_{0} \cdot \exp \left( { - \frac{{{\text{TE}}}}{{T_{2} }}} \right) \cdot \left( {1 - p \cdot \exp \left( { - \frac{{{\text{TI}}}}{{T_{1} }}} \right) + \exp \left( { - \frac{{{\text{TR}}}}{{T_{1} }}} \right)} \right)} \right)$ [34] using MATLAB 2018b (The MathWorks, Inc., Natick, MA, USA). The factor *p* was fitted to account for *B*_1_ errors originating from imperfect 180° inversion pulses.

DTI data were analyzed with FSL’s DTIFIT (FSL v6.1). Distortions induced by eddy currents and head motion in the living subjects were corrected by FSL’s EDDY_CORRECT [35] prior to registering to IR-SE using FLIRT.

### Statistical analysis

The MRI parameters were averaged for each tissue type using the software MATLAB 2018b (The MathWorks, Inc., Natick, MA, USA). The temperature dependence of each MRI parameter was assessed by fitting a linear model to the data, as proposed by Nelson et al. [11]. Further, 95% confidence intervals of the linear fits were determined using MATLAB. A Pearson’s *p* value ≤ 0.05 was interpreted as statistically significant. To detect physiological processes, which might occur during the course of death and further affect the MRI parameters, the fits were performed once including and once excluding the in vivo data. To reflect these possible non-temperature contributions, the percentage difference between the measured mean in vivo value and the value at 36.5 °C predicted by the model using solely post mortem data is shown in Tables 1 and 2 (Δ*) based on the measured mean in vivo value taken as 100%.

**Table 1:** Fitted linear models (*y* = *a* + *bx*) with (w/) and without (w/o) in vivo data for *T*_1_, *T*_2_, *T*_2_*, MD and FA, and differentiated for white matter (top rows), cerebral cortex (middle rows) and deep gray matter (lower rows), respectively

	*a* (*T*_1_ − *T*_2_*, MD, FA: ms, mm^2^/s, a.u.)	*b* (*T*_1_ − *T*_2_*, MD, FA: ms/°C, mm^2^/(s °C), a.u./°C)	*R*^2^	*p*	Δ* [%]	Δ** [%]
**White matter**						
*T*_1_						
w/ invivo	581.5 ± 35.2	0.0 ± 1.7	− 0.03	0.514	6.6	
w/o invivo	566.9 ± 64.9	1.2 ± 4.7	− 0.05	0.637		− 6.6
*T*_2_						
w/ invivo	**105.2** ± **3.3**	− **0.5** ± **0.2**	**0.73**	< **0.001**	− 1.0	
w/o invivo	**105.4** ± **5.6**	− **0.6** ± **0.4**	**0.41**	**0.005**		21.6
*T*_2_*						
w/ invivo	**31.7** ± **3.0**	**0.4** ± **0.1**	**0.63**	< **0.001**	− 22.5	
w/o invivo	36.1 ± 4.1	0.1 ± 0.3	− 0.06	0.733		− 4.6
MD						
w/ invivo	− **4.1E** − **05** ± **7.2E − 05**	**1.6E − 05** ± **3.2E − 06**	**0.85**	< **0.001**	− 56.6	
w/o invivo	**1.0E − 04** ± **4.2E − 05**	**4.3E − 06** ± **2.9E − 06**	**0.45**	**0.003**		− 54.3
FA						
w/ invivo	3.9E − 01 ± 3.1E − 02	4.63E − 04 ± 1.4E − 03	− 0.02	0.422	− 1.9	
w/o invivo	4.0E − 01 ± 2.3E − 02	7.2E − 05 ± 1.0E − 03	− 0.07	0.846		− 0.6
**Cerebral cortex**						
*T*_1_						
w/ invivo	**902.8** ± **73.1**	**3.8** ± **3.6**	**0.21**	**0.024**	14.9	
w/o invivo	**846.4** ± **115.7**	**8.7** ± **8.4**	**0.19**	**0.050**		− 24.3
*T*_2_						
w/ invivo	124.8 ± 10.6	− 0.5 ± 0.5	0.14	0.055	1.9	
w/o invivo	124.3 ± 18.7	− 0.5 ± 1.4	0.03	0.252		15.3
*T*_2_*						
w/ invivo	**32.5** ± **3.4**	**0.3** ± **0.2**	**0.34**	**0.004**	− 17.4	
w/o invivo	35.6 ± 5.5	0.03 ± 0.41	− 0.07	0.838		− 3.1
MD						
w/ invivo	− **1.8E − 05** ± **8.2E − 05**	**2.2E − 05** ± **3.5E − 06**	**0.87**	< **0.001**	− 47.4	
w/o invivo	**1.4E − 04** ± **6.8E − 05**	**7.7E − 06** ± **5.0E − 06**	**0.48**	**0.002**		− 58.8
FA						
w/ invivo	**2.1E − 01** ± **2.4E − 02**	− **8.8E − 04** ± **1.3E − 03**	**0.21**	**0.023**	80.7	
w/o invivo	1.8E − 01 ± 2.5E − 02	1.9E − 03 ± 1.8E − 03	0.09	0.143		− 24.3
**Deep gray matter**						
*T*_1_						
w/ invivo	**569.5** ± **36.7**	**4.6** ± **1.7**	**0.68**	< **0.001**	− 2.8	
w/o invivo	**578.2** ± **61.8**	**3.9** ± **4.2**	**0.27**	**0.023**		− 17.6
*T*_2_						
w/ invivo	90.6 ± 5.6	− 0.2 ± 0.3	0.07	0.137	1.8	
w/o invivo	89.6 ± 9.6	− 0.1 ± 0.7	− 0.03	0.475		4.4
*T*_2_*						
w/ invivo	**22.4** ± **3.2**	**0.4** ± **0.2**	**0.62**	< **0.001**	− 24.3	
w/o invivo	26.2 ± 4.6	0.11 ± 0.33	− 0.04	0.507		− 11.5
MD						
w/ invivo	− **4.8E − 06** ± **4.4E − 05**	**1.6E − 05** ± **2.8E − 06**	**0.86**	< **0.001**	− 49.8	
w/o invivo	**1.2E − 04** ± **4.8E − 05**	**5.2E − 06** ± **3.4E − 06**	**0.40**	**0.005**		− 53.6
FA						
w/ invivo	**4.3E − 01** ± **4.4E − 02**	− **2.6E − 03** ± **2.2E − 03**	**0.27**	**0.011**	48.7	
w/o invivo	3.8E − 01 ± 5.6E − 02	1.8E − 03 ± 3.9E − 03	− 0.03	0.490		− 12.9

95% confidence intervals of the linear fit parameters *y*-intercept (*a*) and slope (*b*) and the adjusted *R*^2^ value and the *p* value are shown. The percentage differences between the measured mean in vivo values and the predicted values at 36.5 °C based on the post mortem data (Δ*), taking the measured mean in vivo values as 100% are indicated. The percentage differences between the maximum temperature difference of 4 and 36.5 °C (Δ**) taking the value at 36.5 °C as 100% are listed based on the fit using solely post mortem data

**Table 2:** Fitted linear models (*y* = *a* + *bx*) with (w/) and without (w/o) in vivo data for *T*_1_, *T*_2_, *T*_2_*, MD and FA, and differentiated for the additionally investigated deep gray matter subregions

	*a* (*T*_1_ − *T*_2_*, MD, FA: ms, mm^2^/s, a.u.)	*b* (*T*_1_ − *T*_2_*, MD, FA: ms/°C, mm^2^/(s °C), a.u./°C)	*R*^2^	*p*	Δ* [%]	Δ** [%]
**Globus pallidus**						
*T*_1_						
w/ invivo	**524.6** ± **17.3**	**3.0** ± **0.7**	**0.78**	< **0.001**	− 4.7	
w/o invivo	**539.6 ± 23.2**	**1.8 ± 1.6**	**0.32**	**0.013**		− 9.8
*T*_2_						
w/ invivo	80.7 ± 6.5	− 0.1 ± 0.3	− 0.01	0.379	1.5	
w/o invivo	80.4 ± 10.5	− 0.1 ± 0.8	− 0.07	0.786		4.5
*T*_2_*						
w/ invivo	**16.6** ± **3.4**	**0.4** ± **0.2**	**0.64**	< **0.001**	− 32.6	
w/o invivo	21.0 ± 4.3	0.1 ± 0.3	− 0.03	0.477		− 8.6
MD						
w/ invivo	**3.9E − 05** ± **5.5E − 05**	**1.1E − 05** ± **2.4E − 06**	**0.80**	< **0.001**	− 51.5	
w/o invivo	1.3E − 04 ± 5.8E − 05	2.5E − 06 ± 4.2E − 06	0.13	0.093		− 36.4
FA						
w/ invivo	**5.0E − 01** ± **3.6E − 02**	− **2.17E − 03** ± **1.7E − 03**	**0.22**	**0.021**	12.0	
w/o invivo	4.8E − 01 ± 3.9E − 02	− 7.1E − 04 ± 2.8E − 03	− 0.06	0.740		5.1
**Putamen**						
*T*_1_						
w/ invivo	**464.8** ± **25.9**	**9.2** ± **1.2**	**0.94**	< **0.001**	− 9.7	
w/o invivo	**500.6** ± **33.4**	**6.5** ± **2.1**	**0.76**	< **0.001**		− 28.6
*T*_2_						
w/ invivo	76.6 ± 6.0	0.0 ± 0.4	− 0.05	0.914	− 1.2	
w/o invivo	76.7 ± 9.9	0.0 ± 0.8	− 0.05	0.569		1.0
*T*_2_*						
w/ invivo	**18.4** ± **4.1**	**0.5** ± **0.2**	**0.53**	< **0.001**	− 26.7	
w/o invivo	22.2 ± 6.0	0.15 ± 0.44	− 0.04	0.548		− 17.3
MD						
w/ invivo	**4.3E − 06** ± **3.8E − 05**	**1.5E − 05** ± **1.7E − 06**	**0.86**	< **0.001**	− 50.0	
w/o invivo	**1.2E − 04** ± **5.2E − 05**	**5.1E − 06** ± **3.7E − 06**	**0.43**	**0.003**		− 54.1
FA						
w/ invivo	**4.7E − 01** ± **5.5E − 02**	− **5.6E − 03** ± **2.8E − 03**	**0.53**	< **0.001**	85.2	
w/o invivo	4.0E − 01 ± 3.4E − 02	6.2E − 05 ± 6.4E − 04	0.00	0.868		− 0.5
**Caudate nucleus**						
*T*_1_						
w/ invivo	**535.7** ± **50.8**	**5.8** ± **2.2**	**0.69**	< **0.001**	− 1.0	
w/o invivo	**539.1** ± **86.4**	**5.6** ± **5.4**	**0.29**	**0.018**		− 24.4
*T*_2_						
w/ invivo	**91.8** ± **4.7**	− **0.3** ± **0.3**	**0.29**	**0.008**	− 4.2	
w/o invivo	93.1 ± 7.7	− 0.4 ± 0.6	0.13	0.095		17.4
*T*_2_*						
w/ invivo	**25.8** ± **4.1**	**0.5** ± **0.2**	**0.52**	< **0.001**	− 26.1	
w/o invivo	30.0 ± 5.9	0.08 ± 0.44	− 0.06	0.663		− 8.0
MD						
w/ invivo	− **1.5E − 05** ± **8.3E − 05**	**1.7E − 05** ± **4.2E − 06**	**0.85**	< **0.001**	− 51.8	
w/o invivo	**1.3E − 04** ± **6.3E − 05**	**5.0E − 06** ± **4.3E − 06**	**0.34**	**0.011**		− 52.4
FA						
w/ invivo	**4.6E − 01** ± **6.0E − 02**	− **4.2E − 03** ± **3.1E − 03**	**0.37**	**0.003**	54.9	
w/o invivo	4.1E − 01 ± 4.0E − 02	1.3E − 04 ± 1.7E − 03	− 0.07	0.825		− 1.0
**Thalamus**						
*T*_1_						
w/ invivo	**566.0** ± **52.4**	**4.4** ± **2.4**	**0.54**	< **0.001**	− 5.6	
w/o invivo	584.7 ± 90.5	3.0 ± 6.0	0.10	0.119		− 13.9
*T*_2_						
w/ invivo	**101.6** ± **6.3**	− **0.4** ± **0.3**	**0.17**	**0.039**	3.9	
w/o invivo	100.2 ± 10.9	− 0.2 ± 0.8	− 0.01	0.373		8.1
*T*_2_*						
w/ invivo	**25.7** ± **3.2**	**0.4** ± **0.1**	**0.61**	< **0.001**	− 21.8	
w/o invivo	29.3 ± 4.5	0.11 ± 0.33	− 0.03	0.444		− 10.5
MD						
w/ invivo	− **4.0E − 06** ± **5.5E − 05**	**1.7E − 05** ± **4.1E − 06**	**0.86**	< **0.001**	− 47.9	
w/o invivo	**1.3E − 04** ± **5.5E − 05**	**5.8E − 06** ± **3.8E − 06**	**0.38**	**0.007**		− 55.4
FA						
w/ invivo	4.0E − 01 ± 5.0E − 02	− 1.1E − 03 ± 2.5E − 03	0.02	0.264	53.3	
w/o invivo	3.4E − 01 ± 6.8E − 02	4.0E − 03 ± 4.7E − 03	0.09	0.132		− 26.5

95% confidence intervals of the linear fit parameters *y*-intercept (*a*) and slope (*b*) and the adjusted *R*^2^ value and the *p* value are shown. The percentage differences between the measured mean in vivo value and the predicted values at 36.5 °C based on the post mortem data (Δ*), taking the measured mean in vivo values as 100% are indicated. The percentage differences between the maximum temperature difference of 4 and 36.5 °C (Δ**) taking the value at 36.5 °C as 100% are listed based on the fit using solely post mortem data

Furthermore, the percentage differences of the MRI parameters at maximum temperature difference between 4 and 36.5 °C (Δ**) was further evaluated based on the fit using solely post mortem data. This difference was computed based on the respective value at 36.5 °C taken as 100%. This percentage difference reflects the contribution of temperature on the in situ post mortem MRI parameters.

## Results

**Fig. 3:**
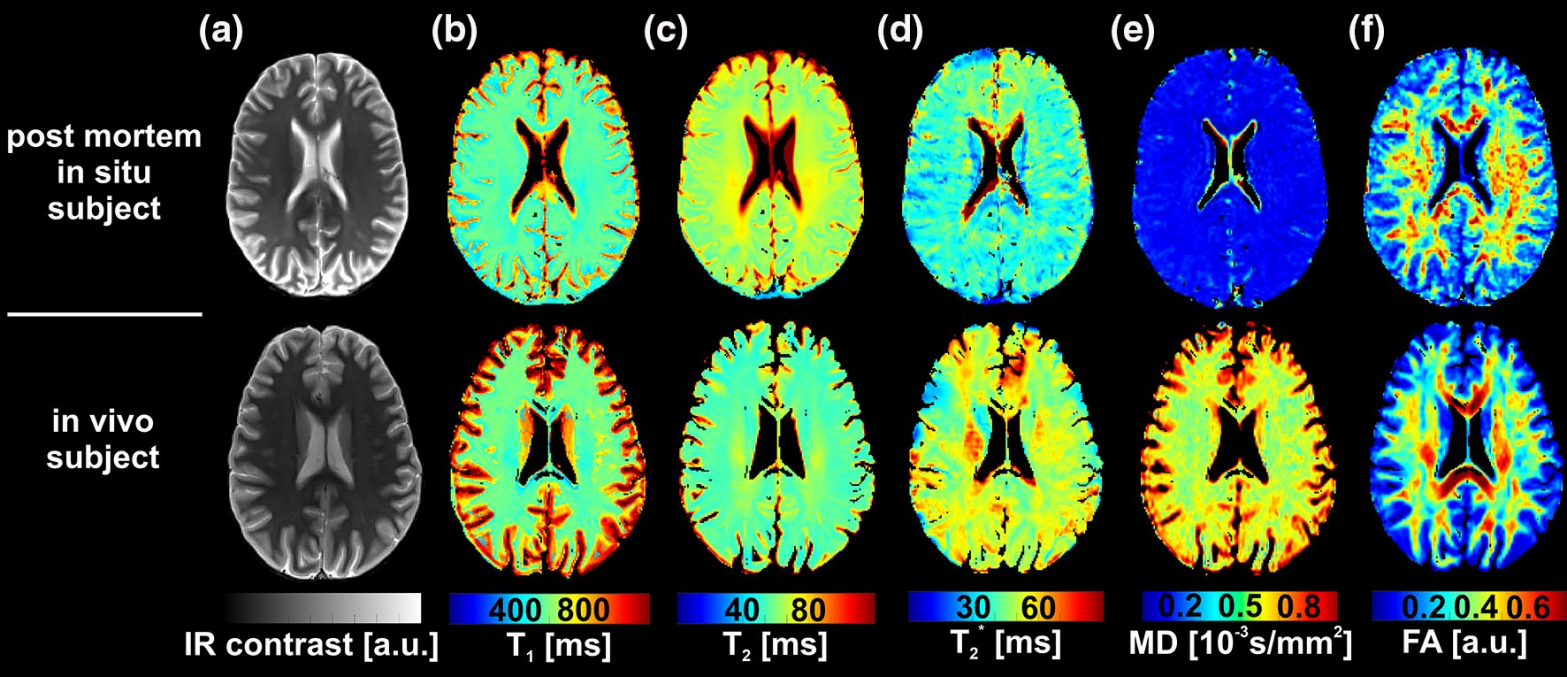
Representative IR-contrast (TI = 200 ms) (**a**), *T*_1_ (**b**), *T*_2_ (**c**), *T*_2_* (**d**), MD (**e**), FA (**f**) maps of one post mortem subject with a brain temperature of 5.6 °C (top row) and one in vivo subject (bottom row)

The deceased revealed an average brain temperature of 12.7 ± 5.7 °C (ranging from 5.6 to 28.1 °C) and an average PMI at the time of the MRI scan of 31.1 ± 16.5 h. Representative *T*_1_, *T*_2_, *T*_2_*, MD, FA maps of one post mortem subject and one in vivo subject are shown in Fig. 3.

**Fig. 4:**
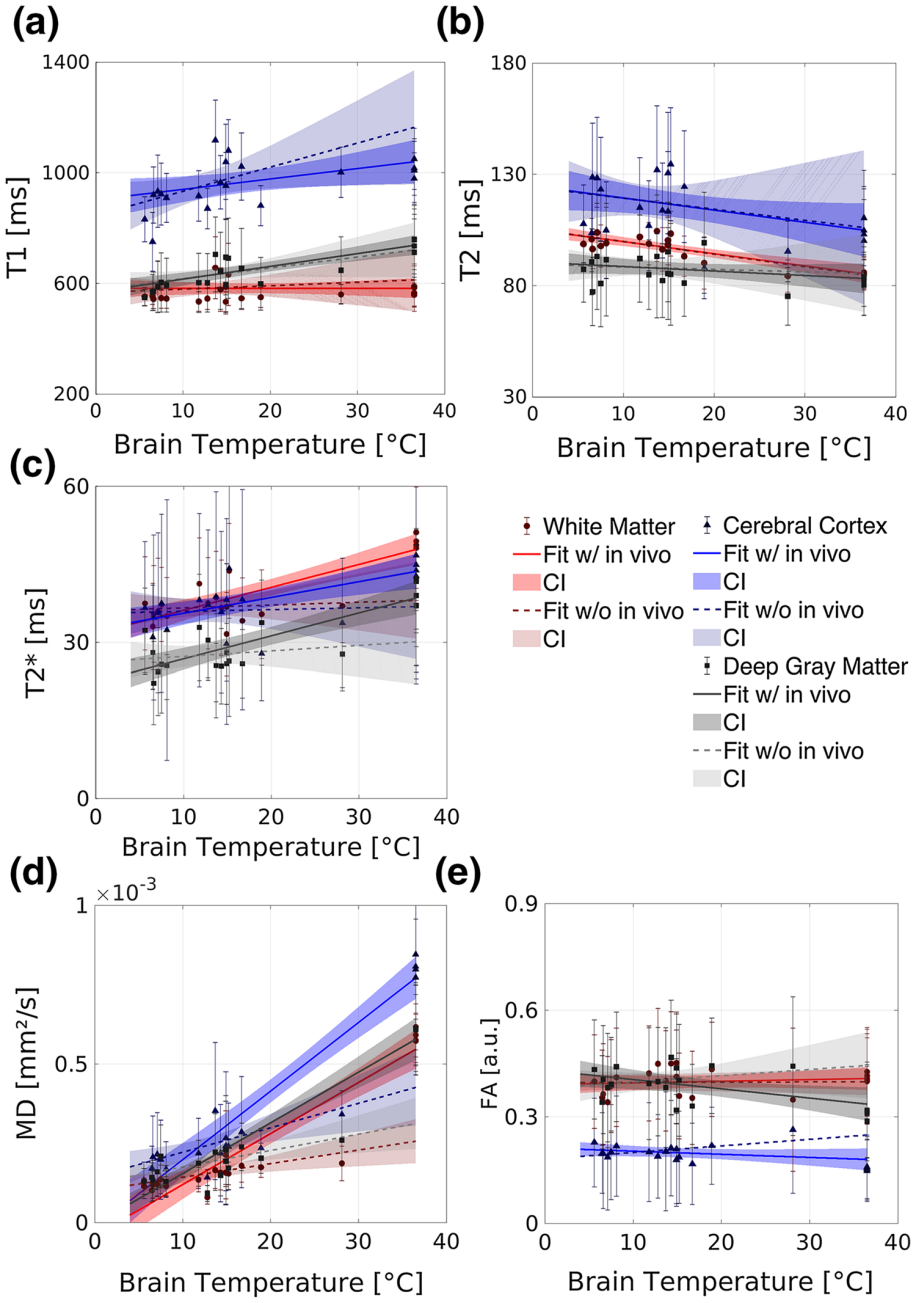
The MRI parameters *T*_1_, *T*_2_, *T*_2_*, MD and FA (**a**–**e**) as a function of the brain temperature differentiated for cerebral cortex (blue triangles), deep gray matter (gray squares) and white (red dots) matter. Error bars represent standard deviation. The corresponding linear fits are shown including the in vivo values (solid line) and excluding the in vivo values (dashed line), respectively. The dark shaded areas indicate the 95% CI of the fits including the in vivo values of cerebral cortex (blue), deep gray matter (gray) and white (red) matter. The light shaded areas indicate the 95% CI of the fits excluding the in vivo values of cerebral cortex (blue), deep gray matter (gray) and white matter (red)

**Fig. 5:**
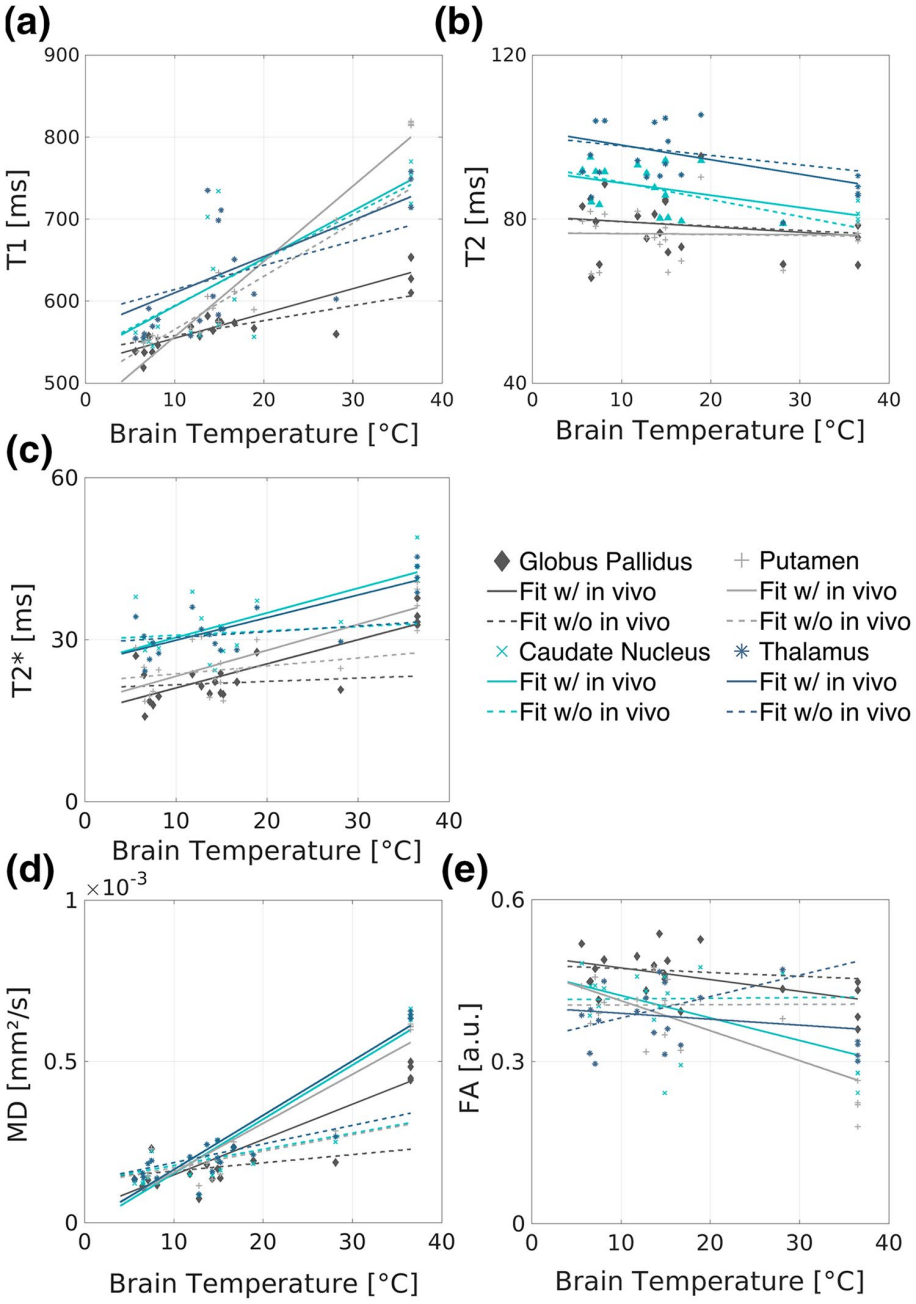
The MRI parameters *T*_1_, *T*_2_, *T*_2_*, MD and FA (**a**–**e**) as a function of the brain temperature differentiated for globus pallidus (dark gray diamonds), putamen (light gray plus signs), caudate nucleus (cyan crosses), and thalamus (dark gray-blue asterisks). The corresponding linear fits including the in vivo values (solid line) and excluding the in vivo values (dashed line) are shown

Table 1 summarizes the temperature effects on all MRI parameters (*T*_1_, *T*_2_, *T*_2_*, MD, FA) for white matter (WM), the cerebral cortex and deep gray matter based on the fitted linear models once with and once without in vivo data. Moreover, Table 2 presents the temperature effect on all MRI parameters for the additionally analyzed deep gray matter substructures, again based on the fitted linear models once with and once without in vivo data. The graphs revealing each MRI parameter as a function of the brain temperature with both fits are shown for white matter, the cerebral cortex and deep gray matter in Fig. 4, as well as for the deep gray matter substructures in Fig. 5.

*T*_1_ of all gray matter (GM) regions significantly increases with increasing temperature based on the fit including the in vivo values (cerebral cortex: *p* = 0.024, deep gray matter regions: *p* < 0.01) and shows the greatest slope of the linear fit of all investigated relaxations parameters. The slope values are similar for the thalamus and the globus pallidus, while a greater slope value is observed in the putamen. *T*_1_ of WM as well as *T*_2_ of the cerebral cortex and deep gray matter do not show any temperature dependence, while *T*_2_ of WM decreases significantly (*p* < 0.01) with increasing brain temperature, independently of the in vivo data. Significant positive correlations (*p* < 0.01) between the brain temperature and *T*_2_* were found in WM, as well as in the cerebral cortex and deep gray matter regarding the fit including the in vivo data. Similar slope values have been found in all deep gray matter regions investigated. The MD increases significantly (*p* < 0.01) with increasing temperature in WM and GM irrespective of including in vivo data or not. FA does not show a significant correlation with temperature in WM, while FA of the cerebral cortex, globus pallidus, caudate nucleus and putamen significantly (*p* < 0.01) correlate with temperature in case the fit included the in vivo data.

The percentage differences between the measured mean in vivo values and the predicted values at 36.5 °C based on the model using solely post mortem data is further shown in Tables 1 and 2 (Δ*). The largest percentage difference Δ* is observed in gray matter regions for the parameter FA (50–80%) and in all regions for MD (~ − 50%).

The last column in Tables 1 and 2 indicates the relative difference between the MRI parameter fit at in vivo brain temperature (36.5 °C) and at minimum brain temperature (4 °C), using the model-based solely on post mortem data (Δ**). The largest Δ** ranging from − 36.4 to − 58.8% was observed for MD in both WM and GM. In WM, *T*_1_, *T*_2_* and FA showed only minimal changes of − 6.6, − 4.6 and − 0.6%, respectively, while for *T*_2_, a difference of 21.6% was obtained. In GM, the changes of *T*_1_, *T*_2,_ *T*_2_* and FA ranged from − 28.6 to 17.4%.

## Discussion

The experimental set-up of this study allowed the investigation of the effect of brain temperature on post mortem quantitative brain MRI. By performing MRI in situ post mortem, this study overcame the limitations of tissue extraction and fixation of ex-situ studies. Additionally, by directly measuring the brain temperature, the issue of having different cooling rates for the body core and the brain faced by previous studies [3, 5–10, 16, 17] was avoided. Moreover, this study enabled the comparison of in situ post mortem and in vivo MRI parameters based on the same experimental set-up.

The longitudinal relaxation rate *T*_1_ of the cerebral cortex and the deep gray matter revealed significant positive correlations as proposed by the FETS model [11]. *T*_1_ of the cerebral cortex revealed the greatest slope value compared to all other gray matter regions based on post mortem conditions. Furthermore, the putamen had the greatest slope value of the deep gray matter subregions, which is in agreement with the findings in the basal ganglia of Birkl et al. [4]. The detected slopes in the cerebral cortex were lower compared to the findings proposed by Birkl et al. [4] and higher compared to the findings of Zech et al. [5]. This could be explained by the differences in the experimental set-up: as this study conducted in situ measurements and performed brain temperature measurements, a direct comparison is difficult as Zech et al. [5] measured the core temperature in the esophagus and Birkl et al. [4] measured the relaxation times of extracted brain tissue at different temperatures by controlled heating on a smaller cohort. Due to restrictions in the present set-up of this study, subjects could not be measured at different temperatures as performed by Birkl et al. [4]. This prevented the evaluation of the role of individual iron and myelin concentrations in different brain regions affecting *T*_1_ in this study [36]. However, by pooling the voxels from across the entire brain in this study, intra-subject influences of tract-specific differences in *T*_1_ could be reduced and intrinsic differences in iron or myelin contents should have averaged out. The resulting *T*_1_ values in this work showed little temperature dependence in WM compared to GM, which might be due to the limited molecular mobility in the intracellular cytoplasm of the myelinated axons or due to the smaller water content in white compared to gray matter influencing the longitudinal relaxation [37, 38]. Therefore, no strong impact of the temperature-induced decrease of molecular mobility on *T*_1_ in WM is observed. As compared to literature, this study observed reduced in vivo *T*_1_ values, although the fit accounted for *B*_1_ imperfections [39–43]. This might originate from methodological discrepancies in the setup of the MRI sequences, or the limited in vivo sample size examined here. Based on the resulting positive Δ* percentage differences, increased *T*_1_ values were obtained in the white matter and cerebral cortex by the sole post mortem fit at 36.5 °C compared to the measured mean in vivo values. This might be attributed to prolonged *T*_1_ values in the older post mortem subjects in white matter [44] and in the cerebral cortex [45]. The significant linear relations and the large Δ** percentage differences of *T*_1_ in the cerebral cortex and the deep gray matter prove the importance of correcting *T*_1_ in gray matter for the temperature by normalizing the values to the same temperature. Thereby, the comparison of quantitative *T*_1_ values among subjects with different brain temperatures, as well as between in vivo and post mortem subjects, could become feasible.

Although the *T*_2_ Δ* percentage differences indicate no strong non-temperature effects, a possible influence of further smaller physiological post mortem effects cannot be ruled out. A temperature effect on *T*_2_ was predicted by the FETS model (− 0.27% per 1 °C in 5% bovine albumin solution) [11]. The similar effect was also observed in WM as well as in the cerebral cortex in this study. However, *T*_2_ only decreased significantly (*p* < 0.01) with increasing temperatures for WM. Non-significant temperature dependencies in the cerebral cortex of *T*_2_ were also observed by previous studies [4, 6]. In deep gray matter, the caudate nucleus and the thalamus solely revealed significant temperature dependencies in case in vivo values were included in the fit. Nevertheless, the increased age of the post mortem subjects compared to the in vivo cohort might have affected *T*_2_ in these regions (by 3.2% and 6.1%, respectively) and therefore, prevents a conclusive statement on the temperature effect based on the fit including in vivo conditions [46]. The significant temperature effect of WM observed in this study revealed a three times larger slope value as compared to previous studies. However, a direct comparison is not feasible, as these studies did not reveal significant temperature dependencies. Based on the significant linear relation and the large Δ** percentage difference observed in WM *T*_2_, a temperature correction of *T*_2_ in WM is crucial to compare *T*_2_ in WM among different brain temperatures and can be achieved with the relation found.

Positive significant correlations can only be observed between the temperature and *T*_2_* in all observed regions in case in vivo values are included in the fit. Notably, the in vivo values revealed smaller WM *T*_2_* variations compared to post mortem conditions, which might be attributed to the decreased *T*_2_* fiber orientation dependency post mortem [47, 48]. The slight positive correlation found in the deep gray matter structures based on the model including the in vivo data may be attributed to the temperature-dependent iron-induced susceptibility changes [49, 50]. Further, shorter *T*_2_* values were predicted by the sole post mortem temperature model at 36.5 °C compared to the measured mean in vivo values (caudate nucleus: − 10.9%, putamen: − 19.5%, globus pallidus: − 7.7%). This may be attributed to the increased age of the post mortem cohort compared to the in vivo subjects (31.5 years on average). According to Sedlacik et al. [46], *T*_2_* is reduced by 12.7% in the basal ganglia for an age difference of 31.5 years, which is in accordance with the results of this study and therefore might contribute to the observed difference between both fits (including and excluding in vivo conditions). In combination with different states of autolysis between in vivo and post mortem subjects, the analysis of the temperature effect including the in vivo values in the deep gray matter structures is limited. No significant temperature effects were found in deep gray matter *T*_2_* based solely on post mortem values, which may be attributed to the limited number of subjects and their inter-subject variations of myelin and iron concentrations additionally affecting *T*_2_*. Together with the limited possibility of this study to investigate the role of myelin and iron contents, no conclusive statement can be drawn on the effect of different iron concentrations in different brain regions on the temperature effect [36, 51, 52].

The temperature effect on MD assessed including the in vivo cases revealed a significantly different fitted linear model compared to the temperature effect based on post mortem cases only. The large and negative Δ* percentage differences for MD indicate the existence of other post mortem effects besides the temperature, which cause a decrease of MD in brain tissue of 50% immediately after death. Similar MD decreases were observed by Scheurer et al. [3] between post mortem and in vivo conditions. Albeit the increased age of the post mortem compared to the in vivo subjects would lead to a reduction of MD, ageing cannot explain the entire difference between both fits (max. 24%, 22% decrease in WM and GM, respectively) [53]. This immediate reduction may be attributed to the cessation of circulation and subsequent loss of perfusion, as well as ceased metabolic activities post mortem, which lead to reduced intra- and extracellular diffusion [54–56]. Moreover, the largest Δ** percentage differences of all MRI parameters were observed in MD of WM, cerebral cortex and deep gray matter revealing values of − 54.3%, − 58.8% and − 53.6%, respectively. Thus, the temperature has a clear effect on MD in both, WM and gray matter brain tissues (*p* < 0.01). These findings are in agreement with the Einstein derivation of the Brownian motion, which suggests a linear relationship between diffusion and temperature [57]. Furthermore, a higher slope was observed in the cerebral cortex compared to WM, thereby indicating a stronger temperature dependence in the cerebral cortex. This might be caused by the lower water content and the limited molecular mobility in WM [58]. Thus, based on the obtained Δ* and Δ** percentage differences, it is important to not only correct MD values for the temperature, but also for the diffusion loss after death to compare MD between in vivo and post mortem subjects.

To compare MD between in vivo and post mortem subjects, it is important to not only correct MD values for the temperature, but also for the diffusion loss after death.

Neither significant post mortem changes, nor a temperature dependency was observed for FA in WM based on the Δ* percentage differences. These findings are in accordance with the results found by Scheurer et al. [3]. In contrast, a significant (*p* < 0.05) temperature dependency was found for FA in the cerebral cortex and deep gray matter based on the fit including the in vivo subjects, albeit no temperature effect on the fractional anisotropy was expected, as it constitutes an entirely structural parameter. Thus, the significance found in GM regions may rather occur due to a post mortem structural change in GM, such as post mortem tissue decomposition that might correlate with temperature. It remains unclear how possible ageing effects influenced our results, as the ageing effect on GM FA was not yet examined in literature and existing publications in WM showed controversial results [53, 59].

### Limitations

The limitations of this study include the different states of autolysis, the already discussed effect of ageing, changes in myelin and iron concentrations, and the small sample size, which limits statistical validity (e.g. non-uniform age and brain temperature distributions). Due to the MR incompatible setup of the brain temperature probe, this study is further limited by the unconsidered temperature increase of the deceased during the MRI measurement. However, previous studies that were based on assessing the rectal temperature faced the same restriction as the rectal temperature probes were also not MRI compatible. To reduce the bias caused by the temperature increase of the brain during the MRI scan, the MRI sequences in this study were always applied in the same order for all subjects. This led to similar environmental temperatures during all MRI examinations. Furthermore, brain temperature of the deceased could not be measured again after the MRI examination, as this would have required an additional CT-controlled placement of the brain probe. In addition, the post mortem quantitative MRI parameters could not be assessed at brain temperatures higher than 28 °C, due to the time-restricted access to the MRI scanner, leading to an increased storage period of the deceased. The limited maximum temperature of the environment in turn led to a limited brain temperature range of 23 °C of the post mortem subjects.

## Conclusion

This study examined the influence of the brain temperature on the MRI parameters of the brain using in vivo and post mortem subjects based on an identical experimental set-up. Brain temperature has a significant influence on *T*_1_, *T*_2_*, MD and FA in GM and on *T*_2_, *T*_2_*, and MD in WM, indicating the necessity to correct these MRI parameters for temperature when measuring post mortem. The linear models proposed in this study can serve as a temperature correction method for in situ post mortem quantitative brain MRI parameters for the varying brain temperatures of deceased. The temperature correction method will allow the direct comparison of brain MRI parameters among different brain temperatures and between in vivo and post mortem subjects. This represents an important precondition for validating quantitative MRI using deceased in which tissue characterization can be complemented by histology. Therefore, the results of this study can be used for future post mortem validation of in vivo brain imaging techniques.
